# Robust inflammatory breast cancer gene signature using nonparametric random forest analysis

**DOI:** 10.1186/s13058-021-01467-y

**Published:** 2021-09-27

**Authors:** Alaa Zare, Lynne-Marie Postovit, John Maringa Githaka

**Affiliations:** 1 Department of Pediatrics, Faculty of Medicine and Dentistry, University of Alberta, Edmonton, AB Canada; 2 Department of Biomedical and Molecular Sciences, Queen’s University, Kingston, ON Canada; 3 Department of Biochemistry, Faculty of Medicine and Dentistry, University of Alberta, Edmonton, AB Canada

**Keywords:** Breast cancer, IBC, IBC signature, Machine learning, Random forest

## Abstract

Inflammatory breast cancer (IBC) is a rare, aggressive cancer found in all the molecular breast cancer subtypes. Despite extensive previous efforts to screen for transcriptional differences between IBC and non-IBC patients, a robust IBC-specific molecular signature has been elusive. We report a novel IBC-specific gene signature (59 genes; G59) that achieves 100% accuracy in discovery and validation samples (45/45 correct classification) and remarkably only misclassified one sample (60/61 correct classification) in an independent dataset. G59 is independent of ER/HER2 status, molecular subtypes and is specific to untreated IBC samples, with most of the genes being enriched for plasma membrane cellular component proteins, interleukin (IL), and chemokine signaling pathways. Our finding suggests the existence of an IBC-specific molecular signature, paving the way for the identification and validation of targetable genomic drivers of IBC.

**Supplementary Information:**

The online version contains supplementary material available at 10.1186/s13058-021-01467-y.

## Introduction

IBC is a rare form of breast cancer associated with poor prognosis compared to other subtypes, and this is attributed to its therapy resistance and a high metastatic potential [1–3]. Moreover, the majority of IBC patients present with late-stage disease wherein the cancer has spread beyond the primary site [4]. To better diagnose and treat IBC patients, the IBC research community is working on defining an IBC-specific molecular signature. The largest study was published through the establishment of the World IBC Consortium which identified 79 genes, molecular subtype-independent, IBC signature [5]. Shortly after, another 132 genes, subtype-independent, IBC signature was reported [6]. However, both signatures were seen in ~ 16.4% and ~ 25% of breast cancer TCGA samples of primarily non-IBC patients, respectively, signifying low specificity in discriminating IBC from non-IBC samples [5, 7–9]. Nevertheless, thus far a robust tumor cell-intrinsic signature that can define IBC from non-IBC or can stratify IBC patients has remained elusive [8, 9]. Indeed, a recent comparison of existing IBC signatures found minimal or no overlap among the proposed genes and none of the signatures could be validated in an independent dataset [9].

In this report, we reanalyzed publicly available gene expression datasets using the nonparametric machine learning random forest (RF) approach. RF is superior to classic statistical approaches used previously on these datasets because (1) It can handle many predictors at once while assigning each a predictor importance score. (2) It uses bootstrap-aggregated (bagged) decision trees to minimize overfitting, allowing for a robust model that can be validated in independent datasets. By restricting our analysis to microdissected IBC tumor epithelium and matching IBC samples with similar receptor-status to non-IBC samples, we have identified an IBC signature of 59 genes that only misclassified one patient out of a total 106 patients in pre-treatment datasets.

## Methods

### Patients’ samples

All analysis was carried out on MATLAB R2018b (MathWorks). Three microarray datasets were downloaded under accession number GSE45581 [6], GSE5847 [10], and GSE111477 [11]. The Cancer Genome Atlas (TCGA) breast cancer dataset was downloaded from cBioPortal (TCGA Firehose Legacy https://www.cbioportal.org/study/summary?id=brca_tcga). GSE45581 was used for discovery and comprised 20 IBC, 20 non-IBC, and 5 normal microdissected patient epithelium samples. GSE5847 is primarily post-treatment samples dataset, comprised of 13 IBC and 35 non-IBC microdissected patient samples. GSE111477 is a dataset of 33 IBC and 28 non-IBC pre-treatment patient samples comprised primarily of the epithelial tissue.

### Genes signature identification, validation, PAM50 subtyping, and ROR score

**Fig. 1:**
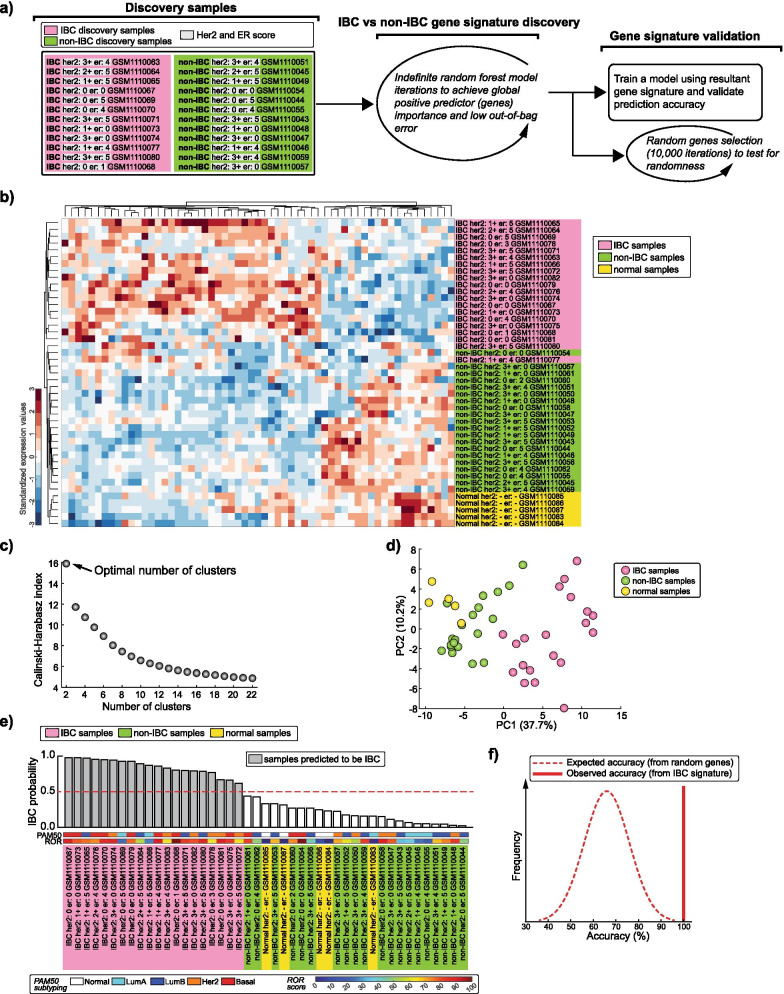
Identification of an IBC-specific gene signature. **a** Left: List of IBC and non-IBC samples used for gene signature discovery (GSE45581 dataset). Row wise matched HER2/ER scores are highlighted and sample accessions numbers (GSM) from gene expression omnibus (GEO) database are indicated. Middle: Strategy for signature discovery. Right: Strategy for signature validation. **b** Unsupervised hierarchical clustering heatmap of all samples (GSE45581 dataset) using the IBC signature genes. **c** The Optimal number of clusters determined by the Caliński–Harabasz criterion. **d** Principal Component Analysis scatter plot using the first and second principal components. **e** Waterfall plot for all samples’ IBC probability score (see Additional file 1: Supp. Methods) validating the signature. The dotted line demarcates the minimum probability score to classify the sample as IBC in the model. PAM50 molecular subtyping and ROR scores are indicated. **f** Distribution of expected accuracy from models trained using random sets of 59 genes (10,000 iterations) compared with the 100% accuracy observed in IBC signature (dotted distribution line versus solid vertical line, respectively)

**Fig. 2:**
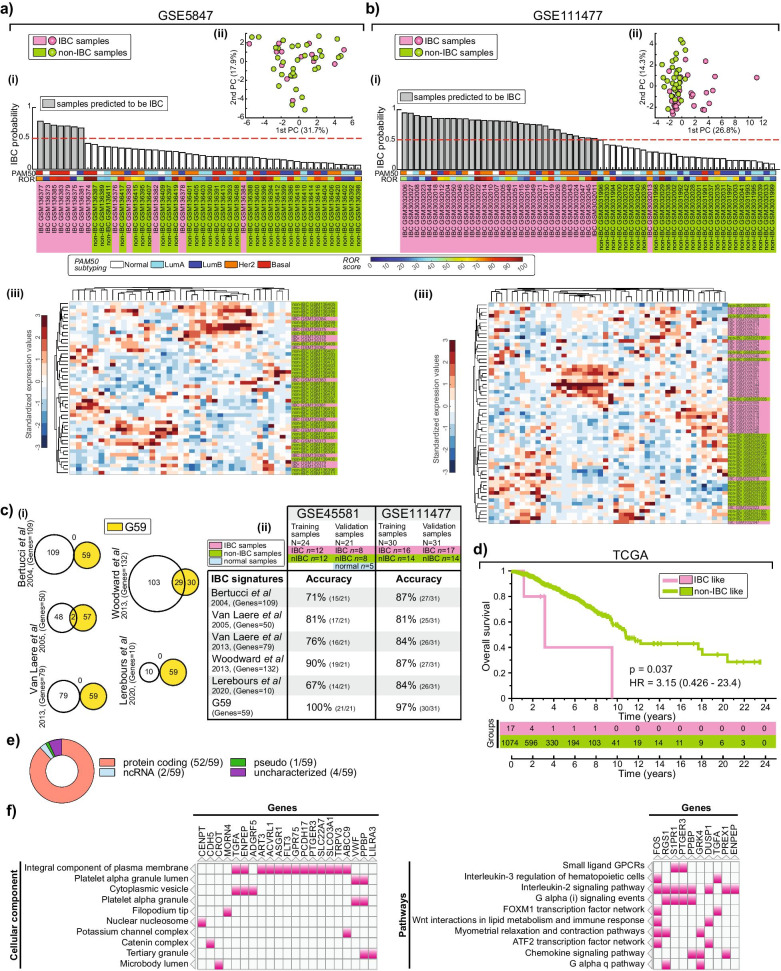
Independent validation of IBC gene signature and its gene ontology/pathway analysis. **a**, **b** Validation of post-treatment samples from GSE5847 dataset and pre-treatment core biopsies samples from GSE111477 dataset, respectively. IBC probability plot, PCA scatter plot and unsupervised hierarchical clustering heatmaps are represented similar to Fig. 1. **c** (i) Venn plots for G59 overlap with 5 previous IBC gene signatures (see Additional file 1: Supp. Methods). (ii) Table indicating the accuracy of the signatures in GSE45581 and GSE111477 datasets (See details in Additional file 1: Supp. Methods). **d** Kaplan–Meier plot log-rank test for G59-predicted IBC like versus non-IBC like samples in TCGA (see details in Additional file 1: Supp. Methods). The p-value, hazard ratio (HR) and the 95% confidence interval of ratio are indicated. **e** Pie chart indicating the proportion of gene types in the signature. ncRNA: non-coding RNA. **f** Clustergrams of top 10 cellular component and pathway analysis of the signature genes, with overlapping genes highlighted (see Additional file 1: Table S3 and S4 for complete list)

IBC-specific signature identification and validation using ensemble of decision trees based bagging is detailed in Additional file 1: Supp. Methods and illustrated in Fig. 1a. For accuracy of 5 previous IBC signatures [Fig. 2c(ii)], PAM50 molecular subtyping (Luminal A, Luminal B, HER2-enriched, Basal-like, and Normal-like) and Risk of recurrence (ROR) computation, see Additional file 1: Supp. Methods.

### Gene ontology and pathway analysis

The IBC signature genes (Additional file 1: Table S1) were subjected to Gene Ontology and Pathway analysis (see Additional file 1: Supp. Methods).

## Results

### Random forest identifies an IBC-specific gene signature

We reanalyzed the gene expression dataset of microdissected epithelial tissues, comprised of 20 IBC, 20 non-IBC, and 5 normal patients [6]. To control for any variability in signature discovery caused by the molecular breast cancer subtypes, we matched both ER and HER2 status of 22/24 samples used for training (Fig. 1a, left, see highlighted ER and HER2 scores). Using the RF approach (Fig. 1a), we derived a potential IBC-specific signature of 59 unique genes (G59, Additional file 1: Table S1).

G59 can comfortably segregate IBC from non-IBC and normal samples in unsupervised hierarchical clustering analysis (Fig. 1b). Caliński-Harabasz criterion on G59 profiles indicated that the samples would best be categorized into two groups: IBC versus non-IBC and normal samples (Fig. 1c). Consistent with this, the first and second principal component scatter plot from the principal component analysis (PCA) of the G59 profiles also separated the IBC samples from the rest (Fig. 1d).

To verify the efficacy of G59, we used RF to model with the 24 training samples (Fig. 1a, left) and subsequently classified all the 45 samples using the resultant trained model. Remarkably, G59 model accurately identified all IBC samples (IBC probability score > 0.5) with no misclassification of non-IBC or normal samples (Fig. 1e). This accuracy was significantly higher than would be expected if the signature was just a random set of genes (Fig. 1f). In addition, G59 prediction was independent of ER/HER2 status, molecular subtypes, and ROR (Additional file 1: Table S2). Thus, G59 is a potential IBC-specific signature that can predict IBC samples in a machine learning RF approach.

### The gene signature is predictive in pre-treatment samples

Prior to Woodward et al. IBC dataset [6], only one other microdissected IBC dataset was available [10]. Unlike the Woodward et al. dataset, whose IBC patient samples were collected from pre-treatment core biopsies, this dataset included 13 IBC patients who had primarily received neoadjuvant chemotherapy prior to sample collection. G59 training model correctly classified 7/13 IBC training epithelium samples, as expected, but misclassified the other 6 validation IBC samples [Fig. 2a(i)]. Inline with this, the signature failed to separate IBC from non-IBC samples in both PCA scatter plot and unsupervised hierarchical clustering analysis [Fig. 2a(ii–iii)]. Next, we tested the G59 training model on an independent dataset comprised of 33 IBC and 28 non-IBC core biopsy pre-treatment samples [11]. A trained model using half of the samples from each category only misclassified 1 out of the 61 samples [Fig. 2b(i)], with both PCA scatter plot and unsupervised hierarchical clustering analysis largely separating IBC from non-IBC samples [Fig. 2b(ii–iii)]. This suggests that the G59 signature is predictive of IBC pre-treatment epithelial tumor while chemotherapy treatment abrogated its predictiveness.

### The gene signature is unique to IBC and is enriched in membrane proteins and interleukin pathways

Next, we compared G59 to 5 previous IBC signatures (See details in Additional file 1: Supp. Methods). 49% (29/59) of the genes overlapped with Woodward et al. [6] 132 gene signature with minimal or no overlap with the rest of the signatures [Fig. 2c(i)]. Using RF approach (detailed in Additional file 1: Supp. Methods), G59 accuracy was significantly higher than all the other signatures [Fig. 2c(ii)]. Given the reported low specificity of these IBC signatures in non-IBC samples [5, 7–9], we tested G59 model on TCGA breast cancer dataset, comprised of primarily non-IBC samples. Only 1.6% of the TCGA samples were classified as IBC-like, suggesting G59 was unique to IBC. Indeed, inline with poor overall survival in IBC patients, Kaplan–Meier analysis revealed a higher risk of death for these IBC-like patients, with a hazard ratio of 3.15 (*p* = 0.037) (Fig. 2d).

Having verified G59 signature in two pre-treatment datasets and shown higher specificity in the TCGA dataset, we performed gene ontology and pathway enrichment analysis of the genes. Protein-coding genes presented 88% (52/59) of the gene set (Fig. 2e), with 25% (13/52) being plasma membrane proteins (Fig. 2f left, Additional file 1: Table S3). While there was no overwhelming enrichment of any specific pathway, IL-2, G-alpha, and chemokine pathways gave the highest gene overlap (8, 4, and 3, respectively) with a significant enrichment (Fig. 2f right, Additional file 1: Table S4).

## Discussion

We have identified a robust gene signature that can characterize IBC from non-IBC with an aim to better understand and potentially develop a tailored treatment regimen for IBC patients. G59 is the first IBC signature to be successfully validated in an independent dataset and shows the highest accuracy (100% (45/45) in GSE45581 and (60/61) 98.4% in GSE111477) in its prediction [9]. This is a significant improvement in accuracy as previous signatures accuracy range between 68 and 88% [5, 8, 9], a range similar to our analysis [Fig. 2c(ii)]. Importantly, G59 shows higher specificity in primarily non-IBC TCGA samples compared to previous signatures [5, 7–9].

The low prediction accuracy in primarily post-treatment tumor samples highlights the fact that chemotherapy induces changes in gene expression [12]. Interestingly, SUM149 and SUM190, the two cell lines used in most of the IBC research [13], were derived from patients who had already received chemotherapy treatment [14]. Our analysis suggests the need for establishing IBC cell lines from untreated patients to fully capture IBC-specific profile.

G59 is a more curated version of the 132 gene list selected by Dr. Woodward [6] for IBC assessment with 49% similarities. Most of the genes in G59 code for membrane proteins, suggesting that IBC cells are highly communicative with the tumor microenvironment, likely playing an essential role in directing their disease progression. The novel implication of IL-2 inflammatory as well as chemokine pathways in IBC (Fig. 2f right) adds to the proposed inflammatory pathways involvement [8, 15].

Our finding highlights the need to integrate contemporary statistical approaches to identify molecular signatures previously missed by traditional statistical methods. Most important, the IBC-specific molecular signature we have identified paves the way for IBC functional studies, validation, and potentially successful therapeutic interventions.

## Supplementary Information

**Additional file 1**. **Supplementary Methods and Tables**. Supplementary Methods details genes signature identification, validation and comparison with other IBC signatures, PAM50 subtyping and ROR scores, Gene ontology and pathway analysis. **Table S1** details gene information for the G59 IBC signature. **Table S2** shows distribution of clinical and molecular features in IBC/non-IBC predicted samples. **Table S3** has cellular components for the G59 IBC signature. **Table S4** has pathways analysis for the G59 IBC signature.

## Data Availability

All datasets used are publicly available and referenced in the methods section. MATLAB code and a standalone graphical user interface software are accessible at https://github.com/maringa780/IBCsignature.

## Abbreviations

IBC: Inflammatory breast cancer
RF: Random forest
IL: Interleukin
ROR: Risk of recurrence
HER2: Human epidermal growth factor receptor 2
ER: Estrogen receptor
