# Supplementary material for: Robust inflammatory breast cancer gene signature using nonparametric random forest analysis
Source: Breast Cancer Res. 2021 Sep 27;23:92. doi: 10.1186/s13058-021-01467-y (PMC8477487; doi:10.1186/s13058-021-01467-y)
Supplement: Supplementary file 1 — Additional file 1. Supplementary Methods and Tables. Supplementary Methods details genes signature identification, validation and comparison with other IBC signatures, PAM50 subtyping and ROR scores, Gene ontology and pathway analysis. Table S1 details gene information for the G59 IBC signature. Table S2 shows distribution of clinical and molecular features in IBC/non-IBC predicted samples. Table S3 has cellular components for the G59 IBC signature. Table S4 has pathways analysis for the G59 IBC signature. [file 13058_2021_1467_MOESM1_ESM.pdf]

SUPPLEMENTARY METHODS

Genes Signature identification, validation, and comparison with other IBC signatures

To identify an IBC specific signature, 12 IBC and 12 non-IBC samples (see Fig. 1a, left) were classified in an ensemble of 5000 decision trees based bagging, using probed genes as predictors. This approach was looped through a random forest (RF) training model, removing genes with zero or negative predictor importance in each iteration (predictor importance computed for every tree, then averaged over the entire ensemble and divided by the standard deviation over the entire ensemble), until all genes left had a positive predictor importance and low out-of-bag error (Fig. 1a, middle). This yielded a potential IBC-specific signature of 59 unique genes (Additional file 1: Table S1), that could be used to discriminate IBC from non-IBC patient samples (Fig. 1a, right). Using the 24 samples and G59 to create a RF model, for each sample in GSE45581 dataset, the probability of it being an IBC sample (IBC probability score) was computed from fractions of observations of the class per tree leaf, averaged across all trees in the ensemble. Similarly, for each dataset, GSE5847 and GSE111477, half of the samples for each class were used for training, and the resultant tree ensemble model applied for IBC probability scoring for all samples. To compute the accuracy of previous IBC signatures(1-5), the aforementioned analysis was repeated using the respective signature genes. To score TCGA dataset, raw data was downloaded from cBioPortal TCGA Firehose Legacy (https://www.cbioportal.org/study/summary?id=brca_tcga) . mRNA expression was combined with GSE45581 dataset through quantile normalization. Using the quantile normalized IBC and non-IBC GSE45581 samples, a RF model was trained and used to score the TCGA samples. MatSurv(6) was used to plot the overall survival KM-plot and compute the log-rank test.

PAM50 subtyping and ROR scores

PAM50 molecular subtyping (Luminal A, Luminal B, HER2-enriched, Basal-like, and Normal-like) and Risk of recurrence (ROR)(7) were computed using Bioconductor package *Genefu*(8).

Gene ontology and pathway analysis

The IBC signature genes (Additional file 1: Table S1) were subjected to Gene Ontology and Pathway analysis scoring using the Enrichr web-based application(9). Cellular components and pathways list were ranked using *p*-values for the significance of overlap in Additional file 1: Table S3 and Table S4, respectively.

REFERENCES

(1) Bertucci F, Finetti P, Rougemont J, Charafe-Jauffret E, Nasser V, Loriod B, et al. Gene expression profiling for molecular characterization of inflammatory breast cancer and prediction of response to chemotherapy. Cancer Res 2004 -12-01;64(23):8558-8565.

(2) Van Laere S, Van der Auwera I, Van den Eynden, Gert G., Fox SB, Bianchi F, Harris AL, et al. Distinct molecular signature of inflammatory breast cancer by cDNA microarray analysis. Breast Cancer Res Treat 2005 -10;93(3):237-246.

(3) Van Laere SJ, Ueno NT, Finetti P, Vermeulen P, Lucci A, Robertson FM, et al. Uncovering the molecular secrets of inflammatory breast cancer biology: an integrated analysis of three distinct affymetrix gene expression datasets. Clin Cancer Res 2013 -09-01;19(17):4685-4696.

(4) Woodward WA, Krishnamurthy S, Yamauchi H, El-Zein R, Ogura D, Kitadai E, et al. Genomic and expression analysis of microdissected inflammatory breast cancer. Breast Cancer Res Treat 2013 -04;138(3):761-772.

(5) Lerebours F, Vacher S, Guinebretiere JM, Rondeau S, Caly M, Gentien D, et al. Hemoglobin overexpression and splice signature as new features of inflammatory breast cancer? J Adv Res 2021 -02;28:77-85.

(6) Creed JH, Gerke TA, Berglund AE. MatSurv: Survival analysis and visualization in MATLAB. Journal of Open Source Software 2020 /02/13;5(46):1830.

(7) Parker JS, Mullins M, Cheang MCU, Leung S, Voduc D, Vickery T, et al. Supervised risk predictor of breast cancer based on intrinsic subtypes. J Clin Oncol 2009 -03-10;27(8):1160-1167.

(8) Gendoo DMA, Ratanasirigulchai N, Schröder MS, Paré L, Parker JS, Prat A, et al. Genefu: an R/Bioconductor package for computation of gene expression-based signatures in breast cancer. Bioinformatics 2016 -04-01;32(7):1097-1099.

(9) Kuleshov MV, Jones MR, Rouillard AD, Fernandez NF, Duan Q, Wang Z, et al. Enrichr: a comprehensive gene set enrichment analysis web server 2016 update. Nucleic Acids Res 2016 July 08;44(W1):90.

Table S1. Gene information for the G59 IBC signature

Gene information of G59, including gene symbol, gene type, gene location, and protein name.

IBC-specific genes signature. Original **GPL6480 Gene Symbol**, **current official Gene Symbol**, **Entrez gene ID** (hyperlinked to respective NCBI webpage), **UniProt Protein Name** and **Gene Information** is provided. Highlighted in yellow are genes whose current Gene symbol is different from previous GPL6480 Gene symbol. Highlighted in cyan are genes overlapping with Woodward *et al* 132 gene signature .

GPL6480 Gene Symbol	Current Gene Symbol	Entrez Gene ID	UniProt Protein Name	Gene Info
LYZL1	LYZL1	84569	Lysozyme-like protein 1	84569 [Gene Symbol: LYZL1] [Locus Tag:] [Chromosome: 10] [Map Location: 10p12.1-p11.23] [Description: lysozyme like 1] [Gene Type: protein-coding]
PCDH17	PCDH17	27253	Protocadherin-17	27253 [Gene Symbol: PCDH17] [Locus Tag:] [Chromosome: 13] [Map Location: 13q21.1] [Description: protocadherin 17] [Gene Type: protein-coding]
CBY3	CBY3	646019	Protein chibby homolog 3	646019 [Gene Symbol: CBY3] [Locus Tag:] [Chromosome: 5] [Map Location: 5q35.3] [Description: chibby family member 3] [Gene Type: protein-coding]
LOC100506670	LOC100506670	100506670	-	-uncharacterized
HECW1	HECW1	23072	E3 ubiquitin-protein ligase HECW1	23072 [Gene Symbol: HECW1] [Locus Tag:] [Chromosome: 7] [Map Location: 7p14.1-p13] [Description: HECT, C2 and WW domain containing E3 ubiquitin protein ligase 1] [Gene Type: protein-coding]
LOC100507642	LOC100507642	100507642	-	100507642 [Gene Symbol: LOC100507642] [Locus Tag:] [Chromosome: 7] [Map Location: 7p22.3] [Description: uncharacterized LOC100507642] [Gene Type: ncRNA]
HIST1H4A	H4C1	8359	Histone H4	8359 [Gene Symbol: H4C1] [Locus Tag:] [Chromosome: 6] [Map Location: 6p22.2] [Description: H4 clustered histone 1] [Gene Type: protein-coding]
TIMD4	TIMD4	91937	T-cell immunoglobulin and mucin domain-containing protein 4	91937 [Gene Symbol: TIMD4] [Locus Tag:] [Chromosome: 5] [Map Location: 5q33.3] [Description: T cell immunoglobulin and mucin domain containing 4] [Gene Type: protein-coding]
LILRA3	LILRA3	11026	Leukocyte immunoglobulin-like receptor subfamily A member 3	11026 [Gene Symbol: LILRA3] [Locus Tag:] [Chromosome: 19] [Map Location: 19q13.4] [Description: leukocyte immunoglobulin like receptor A3] [Gene Type: protein-coding]
FAM106CP	FAM106C	100129396	Protein FAM106C	100129396 [Gene Symbol: FAM106C] [Locus Tag:] [Chromosome: 17] [Map Location: 17p11.2] [Description: family with sequence similarity 106 member C] [Gene Type: ncRNA]
CCDC144A	CCDC144A	9720	Coiled-coil domain-containing protein 144A	9720 [Gene Symbol: CCDC144A] [Locus Tag:] [Chromosome: 17] [Map Location: 17p11.2] [Description: coiled-coil domain containing 144A] [Gene Type: protein-coding]
CCDC144NL	CCDC144NL	339184	Putative coiled-coil domain-containing protein 144 N-terminal-like	339184 [Gene Symbol: CCDC144NL] [Locus Tag:] [Chromosome: 17] [Map Location: 17p11.2] [Description: CCDC144A N-terminal pseudogene] [Gene Type: pseudo]
LOC100130741	LOC100130741	100130741	-	-uncharacterized
PPBP	PPBP	5473	Platelet basic protein	5473 [Gene Symbol: PPBP] [Locus Tag:] [Chromosome: 4] [Map Location: 4q13.3] [Description: pro-platelet basic protein] [Gene Type: protein-coding]
CCDC62	CCDC62	84660	Coiled-coil domain-containing protein 62	84660 [Gene Symbol: CCDC62] [Locus Tag:] [Chromosome: 12] [Map Location: 12q24.31] [Description: coiled-coil domain containing 62] [Gene Type: protein-coding]
UNC93A	UNC93A	54346	Protein unc-93 homolog A	54346 [Gene Symbol: UNC93A] [Locus Tag:] [Chromosome: 6] [Map Location: 6q27] [Description: unc-93 homolog A] [Gene Type: protein-coding]
TRPV3	TRPV3	162514	Transient receptor potential cation channel subfamily V member 3	162514 [Gene Symbol: TRPV3] [Locus Tag:] [Chromosome: 17] [Map Location: 17p13.2] [Description: transient receptor potential cation channel subfamily V member 3] [Gene Type: protein-coding]
CENPT	CENPT	80152	Centromere protein T	80152 [Gene Symbol: CENPT] [Locus Tag:] [Chromosome: 16] [Map Location: 16q22.1] [Description: centromere protein T] [Gene Type: protein-coding]
ZCCHC13	ZCCHC13	389874	Zinc finger CCHC domain-containing protein 13	389874 [Gene Symbol: ZCCHC13] [Locus Tag:] [Chromosome: X] [Map Location: Xq13.2] [Description: zinc finger CCHC-type containing 13] [Gene Type: protein-coding]
LOC100129858	SCOC-AS1	100129858	-	-uncharacterized
SLC22A7	SLC22A7	10864	Solute carrier family 22 member 7	10864 [Gene Symbol: SLC22A7] [Locus Tag:] [Chromosome: 6] [Map Location: 6p21.1] [Description: solute carrier family 22 member 7] [Gene Type: protein-coding]
C1orf95	STUM	375057	Protein stum homolog	375057 [Gene Symbol: STUM] [Locus Tag:] [Chromosome: 1] [Map Location: 1q42.12] [Description: stum, mechanosensory transduction mediator homolog] [Gene Type: protein-coding]
GPR75	GPR75	10936	Probable G-protein coupled receptor 75	10936 [Gene Symbol: GPR75] [Locus Tag:] [Chromosome: 2] [Map Location: 2p16.2] [Description: G protein-coupled receptor 75] [Gene Type: protein-coding]
ASGR1	ASGR1	432	Asialoglycoprotein receptor 1	432 [Gene Symbol: ASGR1] [Locus Tag:] [Chromosome: 17] [Map Location: 17p13.1] [Description: asialoglycoprotein receptor 1] [Gene Type: protein-coding]
S1PR1	S1PR1	1901	Sphingosine 1-phosphate receptor 1	1901 [Gene Symbol: S1PR1] [Locus Tag:] [Chromosome: 1] [Map Location: 1p21.2] [Description: sphingosine-1-phosphate receptor 1] [Gene Type: protein-coding]
FAM162B	FAM162B	221303	Protein FAM162B	221303 [Gene Symbol: FAM162B] [Locus Tag:] [Chromosome: 6] [Map Location: 6q22.1] [Description: family with sequence similarity 162 member B] [Gene Type: protein-coding]
CDH5	CDH5	1003	Cadherin-5	1003 [Gene Symbol: CDH5] [Locus Tag:] [Chromosome: 16] [Map Location: 16q21] [Description: cadherin 5] [Gene Type: protein-coding]
VWF	VWF	7450	von Willebrand factor	7450 [Gene Symbol: VWF] [Locus Tag:] [Chromosome: 12] [Map Location: 12p13.31] [Description: von Willebrand factor] [Gene Type: protein-coding]
GPR116	ADGRF5	221395	Adhesion G protein-coupled receptor F5	221395 [Gene Symbol: ADGRF5] [Locus Tag:] [Chromosome: 6] [Map Location: 6p12.3] [Description: adhesion G protein-coupled receptor F5] [Gene Type: protein-coding]
ABCC9	ABCC9	10060	ATP-binding cassette sub-family C member 9	10060 [Gene Symbol: ABCC9] [Locus Tag:] [Chromosome: 12] [Map Location: 12p12.1] [Description: ATP binding cassette subfamily C member 9] [Gene Type: protein-coding]

ACVRL1	ACVRL1	94	Serine/threonine-protein kinase receptor; Receptor protein serine/threonine kinase; Serine/threonine-protein kinase receptor R3	94 [Gene Symbol: ACVRL1] [Locus Tag:] [Chromosome: 12] [Map Location: 12q13.13] [Description: activin A receptor like type 1] [Gene Type: protein-coding]
SLCO3A1	SLCO3A1	28232	Solute carrier organic anion transporter family member 3A1	28232 [Gene Symbol: SLCO3A1] [Locus Tag:] [Chromosome: 15] [Map Location: 15q26.1] [Description: solute carrier organic anion transporter family member 3A1] [Gene Type: protein-coding]
C17orf100	C17orf100	388327	Uncharacterized protein C17orf100	388327 [Gene Symbol: C17orf100] [Locus Tag:] [Chromosome: 17] [Map Location: 17p13.1] [Description: chromosome 17 open reading frame 100] [Gene Type: protein-coding]
ENPEP	ENPEP	2028	Aminopeptidase; Glutamyl aminopeptidase	2028 [Gene Symbol: ENPEP] [Locus Tag:] [Chromosome: 4] [Map Location: 4q25] [Description: glutamyl aminopeptidase] [Gene Type: protein-coding]
FOLH1	FOLH1	2346	Glutamate carboxypeptidase 2	2346 [Gene Symbol: FOLH1] [Locus Tag:] [Chromosome: 11] [Map Location: 11p11.12] [Description: folate hydrolase 1] [Gene Type: protein-coding]
ART3	ART3	419	Ecto-ADP-ribosyltransferase 3; NAD(P)(+)--arginine ADP-ribosyltransferase	419 [Gene Symbol: ART3] [Locus Tag:] [Chromosome: 4] [Map Location: 4q21.1|4p15.1-p14] [Description: ADP-ribosyltransferase 3 (inactive)] [Gene Type: protein-coding]
TGFA	TGFA	7039	Protransforming growth factor alpha	7039 [Gene Symbol: TGFA] [Locus Tag:] [Chromosome: 2] [Map Location: 2p13.3] [Description: transforming growth factor alpha] [Gene Type: protein-coding]
SPNS2	SPNS2	124976	Protein spinster homolog 2	124976 [Gene Symbol: SPNS2] [Locus Tag:] [Chromosome: 17] [Map Location: 17p13.2] [Description: sphingolipid transporter 2] [Gene Type: protein-coding]
NXN	NXN	64359	Nucleoredoxin	64359 [Gene Symbol: NXN] [Locus Tag:] [Chromosome: 17] [Map Location: 17p13.3] [Description: nucleoredoxin] [Gene Type: protein-coding]
PTGER3	PTGER3	5733	Prostaglandin E2 receptor EP3 subtype	5733 [Gene Symbol: PTGER3] [Locus Tag:] [Chromosome: 1] [Map Location: 1p31.1] [Description: prostaglandin E receptor 3] [Gene Type: protein-coding]
NXNL2	NXNL2	158046	Nucleoredoxin-like protein 2	158046 [Gene Symbol: NXNL2] [Locus Tag:] [Chromosome: 9] [Map Location: 9q22.1] [Description: nucleoredoxin like 2] [Gene Type: protein-coding]
PREX1	PREX1	57580	Phosphatidylinositol 3,4,5-trisphosphate-dependent Rac exchanger 1 protein	57580 [Gene Symbol: PREX1] [Locus Tag:] [Chromosome: 20] [Map Location: 20q13.13] [Description: phosphatidylinositol-3,4,5-trisphosphate dependent Rac exchange factor 1] [Gene Type: protein-coding]
MRPS30	MRPS30	10884	39S ribosomal protein S30, mitochondrial	10884 [Gene Symbol: MRPS30] [Locus Tag:] [Chromosome: 5] [Map Location: 5p12] [Description: mitochondrial ribosomal protein S30] [Gene Type: protein-coding]
WDR65	CFAP57	149465	Cilia- and flagella-associated protein 57	149465 [Gene Symbol: CFAP57] [Locus Tag:] [Chromosome: 1] [Map Location: 1p34.2] [Description: cilia and flagella associated protein 57] [Gene Type: protein-coding]
FLT3	FLT3	2322	Receptor-type tyrosine-protein kinase FLT3; Receptor protein-tyrosine kinase	2322 [Gene Symbol: FLT3] [Locus Tag:] [Chromosome: 13] [Map Location: 13q12.2] [Description: fms related receptor tyrosine kinase 3] [Gene Type: protein-coding]
SEMA3E	SEMA3E	9723	Semaphorin-3E	9723 [Gene Symbol: SEMA3E] [Locus Tag:] [Chromosome: 7] [Map Location: 7q21.11] [Description: semaphorin 3E] [Gene Type: protein-coding]
CCDC71	CCDC71	64925	Coiled-coil domain-containing protein 71	64925 [Gene Symbol: CCDC71] [Locus Tag:] [Chromosome: 3] [Map Location: 3p21.31] [Description: coiled-coil domain containing 71] [Gene Type: protein-coding]
GRK4	GRK4	2868	G protein-coupled receptor kinase 4	2868 [Gene Symbol: GRK4] [Locus Tag:] [Chromosome: 4] [Map Location: 4p16.3] [Description: G protein-coupled receptor kinase 4] [Gene Type: protein-coding]
CROT	CROT	54677	Peroxisomal carnitine O-octanoyltransferase	54677 [Gene Symbol: CROT] [Locus Tag:] [Chromosome: 7] [Map Location: 7q21.12] [Description: carnitine O-octanoyltransferase] [Gene Type: protein-coding]
LOC100507508	LOC100507508	100507508	-	-uncharacterized
MORN4	MORN4	118812	MORN repeat-containing protein 4	118812 [Gene Symbol: MORN4] [Locus Tag:] [Chromosome: 10] [Map Location: 10q24.2] [Description: MORN repeat containing 4] [Gene Type: protein-coding]
DUSP1	DUSP1	1843	Dual specificity protein phosphatase 1	1843 [Gene Symbol: DUSP1] [Locus Tag:] [Chromosome: 5] [Map Location: 5q35.1] [Description: dual specificity phosphatase 1] [Gene Type: protein-coding]
FOS	FOS	2353	Cellular oncogene fos; Proto-oncogene c-Fos	2353 [Gene Symbol: FOS] [Locus Tag:] [Chromosome: 14] [Map Location: 14q24.3] [Description: Fos proto-oncogene, AP-1 transcription factor subunit] [Gene Type: protein-coding]
RGS1	RGS1	5996	Regulator of G-protein signaling 1	5996 [Gene Symbol: RGS1] [Locus Tag:] [Chromosome: 1] [Map Location: 1q31.2] [Description: regulator of G protein signaling 1] [Gene Type: protein-coding]
C8orf4	TCIM	56892	Transcriptional and immune response regulator	56892 [Gene Symbol: TCIM] [Locus Tag:] [Chromosome: 8] [Map Location: 8p11.21] [Description: transcriptional and immune response regulator] [Gene Type: protein-coding]
GLYAT	GLYAT	10249	Glycine N-acyltransferase	10249 [Gene Symbol: GLYAT] [Locus Tag:] [Chromosome: 11] [Map Location: 11q12.1] [Description: glycine-N-acyltransferase] [Gene Type: protein-coding]
PLK5	PLK5	126520	Inactive serine/threonine-protein kinase PLK5	126520 [Gene Symbol: PLK5] [Locus Tag:] [Chromosome: 19] [Map Location: 19p13.3] [Description: polo like kinase 5 (inactive)] [Gene Type: protein-coding]
PSPH	PSPH	5723	Phosphoserine phosphataseO-phosphoserine phosphohydrolase	5723 [Gene Symbol: PSPH] [Locus Tag:] [Chromosome: 7] [Map Location: 7p11.2] [Description: phosphoserine phosphatase] [Gene Type: protein-coding]
SPATA5L1	SPATA5L1	79029	Spermatogenesis-associated protein 5-like protein 1	79029 [Gene Symbol: SPATA5L1] [Locus Tag:] [Chromosome: 15] [Map Location: 15q21.1] [Description: spermatogenesis associated 5 like 1] [Gene Type: protein-coding]

Table S2. Distribution of clinical and molecular features in IBC / non-IBC predicted samples

Samples were dichotomized into predicted IBC and non-IBC samples based on IBC probability score and grouped based on clinical features as well as PAM50 molecular subtyping and subtype based risk of relapse (see Supp. Methods). χ2 distribution p-value was computed for each contigency table.

predicted IBC samples
predicted non-IBC samples

GSE45581 (discovery dataset)

GSE45581-PAM50 molecular subtyping (n = 40)

	Normal	Luminal A	Luminal B	Her2	Basal	
Predicted IBC	0	2	4	6	8	χ2 p-value = 0.2135
Predicted non-IBC	0	3	9	5	3	

GSE45581-ROR score (n = 40)

	Low	Intermediate	High	
Predicted IBC	2	0	18	χ2 p value = 0.0548
Predicted non-IBC	2	5	13	

GSE45581-ER score (n = 40)

	Low (ER 0-2)	High (ER 3-5)	
Predicted IBC	8	12	χ2 p value > 0.9999
Predicted non-IBC	8	12	

GSE45581-Her2 score (n = 40)

	Low (ER 0-2)	High (ER 3-5)	
Predicted IBC	13	7	χ2 p value > 0.9999
Predicted non-IBC	12	8	

GSE111477

GSE111477-PAM50 molecular subtyping (n = 61)

	Normal	Luminal A	Luminal B	Her2	Basal	
Predicted IBC	4	4	5	13	6	χ2 p value = 0.0057
Predicted non-IBC	2	12	10	4	1	

GSE111477-ROR score (n = 61)

	Low	Intermediate	High	
Predicted IBC	5	12	15	χ2 p value = 0.0182
Predicted non-IBC	14	5	10	

Table S3. Cellular component for the G59 IBC signature

Cellular components list with rows ranked using p-values for the significance of overlap.

Cellular component Gene Ontology analysis								
**Term**	**Overlap**	**P-value**	**Adjusted P-value**	**Old P-value**	**Old Adjusted P-value**	**Odds Ratio**	**Combined Score**	**Genes**
integral component of plasma membrane (GO:0005887)	13/1463	1.30E-04	0.005706	0	0	3.94803	35.33668	ACVRL1;ENPEP;FLT3;PTGER3;GPR75;TGFA;TRPV3;ABCC9;ASGR1;PCDH17;ART3;SLCO3A1;SLC22A7
platelet alpha granule lumen (GO:0031093)	2/67	0.014645	0.237796	0	0	11.54136	48.74701	VWF;PPBP
cytoplasmic vesicle (GO:0031410)	3/215	0.021339	0.237796	0	0	5.370011	20.65972	ENPEP;ADGRF5;TGFA
platelet alpha granule (GO:0031091)	2/90	0.025485	0.237796	0	0	8.515009	31.24726	VWF;PPBP
filopodium tip (GO:0032433)	1/10	0.027168	0.237796	0	0	41.02058	147.9085	MORN4
nuclear nucleosome (GO:0000788)	1/13	0.035176	0.237796	0	0	30.7608	102.9683	CENPT
potassium channel complex (GO:0034705)	1/14	0.037831	0.237796	0	0	28.39316	92.97685	ABCC9
catenin complex (GO:0016342)	1/28	0.074256	0.338307	0	0	13.66118	35.52223	CDH5
tertiary granule (GO:0070820)	2/164	0.074852	0.338307	0	0	4.608199	11.94557	PPBP;LILRA3
peroxisomal matrix (GO:0005782)	1/42	0.109327	0.338307	0	0	8.990063	19.89874	CROT
microbody lumen (GO:0031907)	1/42	0.109327	0.338307	0	0	8.990063	19.89874	CROT
endoplasmic reticulum-Golgi intermediate compartment membrane (GO:0033116)	1/47	0.121533	0.338307	0	0	8.01087	16.88346	TGFA
chromosome, centromeric region (GO:0000775)	1/49	0.126369	0.338307	0	0	7.676312	15.8788	CENPT
lytic vacuole membrane (GO:0098852)	2/233	0.134601	0.338307	0	0	3.220453	6.458419	ENPEP;SPNS2
ER to Golgi transport vesicle membrane (GO:0012507)	1/54	0.138346	0.338307	0	0	6.950384	13.74783	TGFA
tertiary granule lumen (GO:1904724)	1/55	0.140722	0.338307	0	0	6.821331	13.37641	PPBP
filopodium (GO:0030175)	1/60	0.152506	0.338307	0	0	6.241682	11.73781	MORN4
ficolin-1-rich granule membrane (GO:0101003)	1/61	0.154843	0.338307	0	0	6.137346	11.44824	LILRA3
bicellular tight junction (GO:0005923)	1/72	0.180143	0.338307	0	0	5.18362	8.884743	CDH5
tertiary granule membrane (GO:0070821)	1/73	0.182406	0.338307	0	0	5.111368	8.6971	LILRA3
peroxisomal part (GO:0044439)	1/75	0.186913	0.338307	0	0	4.972723	8.339813	CROT
COPII-coated ER to Golgi transport vesicle (GO:0030134)	1/75	0.186913	0.338307	0	0	4.972723	8.339813	TGFA
chromosomal region (GO:0098687)	1/76	0.189157	0.338307	0	0	4.906173	8.16964	CENPT
lysosomal membrane (GO:0005765)	2/291	0.190654	0.338307	0	0	2.56656	4.253548	ENPEP;SPNS2
secretory granule lumen (GO:0034774)	2/317	0.216741	0.338307	0	0	2.351602	3.595724	VWF;PPBP
specific granule membrane (GO:0035579)	1/91	0.222102	0.338307	0	0	4.085391	6.146946	LILRA3
peroxisome (GO:0005777)	1/92	0.224251	0.338307	0	0	4.040293	6.040187	CROT
microbody (GO:0042579)	1/92	0.224251	0.338307	0	0	4.040293	6.040187	CROT
centriole (GO:0005814)	1/95	0.230664	0.338307	0	0	3.910757	5.736279	CBY3
endoplasmic reticulum-Golgi intermediate compartment (GO:0005793)	1/95	0.230664	0.338307	0	0	3.910757	5.736279	TGFA
clathrin-coated vesicle (GO:0030136)	1/100	0.241235	0.342398	0	0	3.712308	5.278844	VWF
microtubule organizing center part (GO:0044450)	1/126	0.293948	0.404179	0	0	2.936296	3.595057	CBY3
lysosome (GO:0005764)	2/422	0.323735	0.431647	0	0	1.754268	1.978516	ENPEP;SPNS2
RNA polymerase II transcription factor complex (GO:0090575)	1/147	0.333885	0.432086	0	0	2.511289	2.75478	FOS
specific granule (GO:0042581)	1/160	0.357487	0.449412	0	0	2.304449	2.370485	LILRA3
ficolin-1-rich granule (GO:0101002)	1/184	0.398923	0.487572	0	0	1.999798	1.837789	LILRA3
nuclear chromatin (GO:0000790)	1/253	0.50398	0.599328	0	0	1.447163	0.991622	CENPT
mitochondrial matrix (GO:0005759)	1/308	0.574611	0.665339	0	0	1.184582	0.656332	GLYAT
mitochondrial inner membrane (GO:0005743)	1/341	0.612145	0.690626	0	0	1.06781	0.524066	MRPS30
perinuclear region of cytoplasm (GO:0048471)	1/378	0.65037	0.709993	0	0	0.961195	0.413519	TGFA
nuclear chromosome part (GO:0044454)	1/392	0.663847	0.709993	0	0	0.926115	0.379433	CENPT
nucleoplasm part (GO:0044451)	1/407	0.677721	0.709993	0	0	0.891215	0.3467	CENPT
mitochondrion (GO:0005739)	2/1026	0.780968	0.79913	0	0	0.697266	0.172379	MRPS30;GLYAT
nuclear body (GO:0016604)	1/618	0.822481	0.822481	0	0	0.580107	0.11337	CENPT

Table S4. Pathways analysis for the G59 IBC signature

Pathways analysis list with rows ranked using p-values for the significance of overlap.

BioPlanet pathway analysis								
Term	Overlap	P-value	Adjusted P-value	Old P-value	Old Adjusted P-value	Odds Ratio	Combined Score	Genes
Small ligand GPCRs	2/19	0.001232	0.117835	0	0	44.23529	296.331566	PTGER3;S1PR1
Interleukin-3 regulation of hematopoietic cells	2/20	0.001367	0.117835	0	0	41.77568	275.526768	TGFA;FOS
Interleukin-2 signaling pathway	8/847	0.002086	0.117835	0	0	3.876144	23.9246098	PREX1;ENPEP;RGS1;DUSP1;PTGER3;S1PR1;FOS;PPBP
G alpha (i) signaling events	4/199	0.002182	0.117835	0	0	7.94369	48.6745734	RGS1;PTGER3;S1PR1;PPBP
FOXM1 transcription factor network	2/41	0.005684	0.196055	0	0	19.26076	99.5789646	TGFA;FOS
Wnt interactions in lipid metabolism and immune response	2/45	0.006815	0.196055	0	0	17.46556	87.129734	DUSP1;FOS
Myometrial relaxation and contraction pathways	3/155	0.008922	0.196055	0	0	7.512525	35.4537037	RGS1;GRK4;FOS
ATF2 transcription factor network	2/59	0.011493	0.196055	0	0	13.1665	58.8023751	DUSP1;FOS
Chemokine signaling pathway	3/189	0.015206	0.196055	0	0	6.128722	25.6553397	PREX1;GRK4;PPBP
G alpha q pathway	2/70	0.015912	0.196055	0	0	11.03052	45.6736747	RGS1;GRK4
AP-1 transcription factor network	2/70	0.015912	0.196055	0	0	11.03052	45.6736747	DUSP1;FOS
Beta-oxidation of pristanoyl-CoA	1/8	0.021793	0.196055	0	0	52.74603	201.815026	CROT
Lysosphingolipid and lysophosphatidic acid (LPA) G-protein coupled receptors	1/8	0.021793	0.196055	0	0	52.74603	201.815026	S1PR1
TSP1-induced apoptosis in microvascular endothelial cell	1/8	0.021793	0.196055	0	0	52.74603	201.815026	FOS
Response to elevated platelet cytosolic calcium	2/83	0.02192	0.196055	0	0	9.254135	35.3542477	VWF;PPBP
Prostanoid ligand receptors	1/9	0.024484	0.196055	0	0	46.15046	171.205697	PTGER3
MAP kinase pathway regulation through dual specificity phosphatases	1/9	0.024484	0.196055	0	0	46.15046	171.205697	DUSP1
Activation of the AP-1 family of transcription factors	1/10	0.027168	0.196055	0	0	41.02058	147.908479	FOS
Glycoprotein 1b-IX-V activation signaling	1/10	0.027168	0.196055	0	0	41.02058	147.908479	VWF
Attenuation of GPCR signaling	1/11	0.029845	0.196055	0	0	36.91667	129.642094	GRK4
TGF-beta signaling in gastrointestinal stem cells	1/11	0.029845	0.196055	0	0	36.91667	129.642094	ACVRL1
Transport of organic anions	1/11	0.029845	0.196055	0	0	36.91667	129.642094	SLCO3A1
ERBB1 downstream pathway	2/106	0.034441	0.196055	0	0	7.199202	24.2504942	DUSP1;FOS
Platelet adhesion to exposed collagen	1/13	0.035176	0.196055	0	0	30.7608	102.968257	VWF
Organic anion transporters	1/13	0.035176	0.196055	0	0	30.7608	102.968257	SLC22A7
Organic cation/anion/zwitterion transport	1/13	0.035176	0.196055	0	0	30.7608	102.968257	SLC22A7
G alpha i pathway	2/108	0.035636	0.196055	0	0	7.062656	23.5497447	RGS1;GRK4
Leptin influence on immune response	2/110	0.036846	0.196055	0	0	6.931167	22.8797863	FOS;PPBP
Interleukin-4 regulation of apoptosis	3/267	0.037114	0.196055	0	0	4.300918	14.1662417	S1PR1;FOS;CCDC71
T cell receptor/Ras pathway	1/14	0.037831	0.196055	0	0	28.39316	92.9768536	FOS
Repression of pain sensation by the transcriptional regulator DREAM	1/15	0.040479	0.196055	0	0	26.36376	84.5478253	FOS
Calcium signaling by HBx of hepatitis B virus	1/15	0.040479	0.196055	0	0	26.36376	84.5478253	FOS
Coagulation intrinsic pathway	1/15	0.040479	0.196055	0	0	26.36376	84.5478253	VWF
Eicosanoid ligand-binding G-protein coupled receptors	1/15	0.040479	0.196055	0	0	26.36376	84.5478253	PTGER3
Erythrocyte differentiation pathway	1/15	0.040479	0.196055	0	0	26.36376	84.5478253	FLT3
Amino acid biosynthesis and interconversion (transamination)	1/16	0.04312	0.196055	0	0	24.60494	77.35242	PSPH
B cell survival pathway	1/16	0.04312	0.196055	0	0	24.60494	77.35242	FOS
Selenium metabolism and selenoproteins	1/16	0.04312	0.196055	0	0	24.60494	77.35242	FOS
CD40L signaling pathway	1/16	0.04312	0.196055	0	0	24.60494	77.35242	DUSP1
Other semaphorin interactions	1/16	0.04312	0.196055	0	0	24.60494	77.35242	SEMA3E
G alpha s pathway	2/120	0.043131	0.196055	0	0	6.340582	19.9317377	RGS1;GRK4
GRB2-SOS provides linkage to MAPK signaling for integrins	1/17	0.045753	0.196055	0	0	23.06597	71.1468518	VWF
Renin-angiotensin system	1/17	0.045753	0.196055	0	0	23.06597	71.1468518	ENPEP
Cadmium-induced DNA biosynthesis and proliferation in macrophages	1/17	0.045753	0.196055	0	0	23.06597	71.1468518	FOS
Interleukin-1 signaling pathway	2/125	0.046413	0.196055	0	0	6.081301	18.6706762	DUSP1;FOS
Pertussis toxin-insensitive CCR5 signaling in macrophage	1/18	0.04838	0.196055	0	0	21.70806	65.7467123	FOS
Bone remodeling	1/18	0.04838	0.196055	0	0	21.70806	65.7467123	FOS
TNFR2 signaling pathway	1/18	0.04838	0.196055	0	0	21.70806	65.7467123	DUSP1
Effect of METS on macrophage differentiation	1/18	0.04838	0.196055	0	0	21.70806	65.7467123	FOS
PDGFB signaling pathway	2/129	0.049103	0.196055	0	0	5.888575	17.7471999	S1PR1;FOS
